# Supplementary material for: A vascularized tumoroid model for human glioblastoma angiogenesis
Source: Sci Rep. 2021 Oct 1;11:19550. doi: 10.1038/s41598-021-98911-y (PMC8486855; doi:10.1038/s41598-021-98911-y)
Supplement: Supplementary file 1 — Supplementary Information. [file 41598_2021_98911_MOESM1_ESM.pdf]

A Vascularized Tumoroid Model for Human Glioblastoma Angiogenesis

Agavi Stavropoulou Tatla[1,*], Alexander W Justin[1], Colin Watts[2], and Athina E Markaki[1,+]

[1]Department of Engineering, University of Cambridge, Trumpington Street, Cambridge CB2 1PZ, UK
[2]Division of Neurosurgery, Department of Clinical Neurosciences, University of Cambridge, Addenbrooke's Hospital, Cambridge CB2 0QQ, UK
[2]Present address: Institute of Cancer and Genomic Sciences, College of Medical and Dental Sciences, University of Birmingham, Edgbaston, Birmingham B15 2TT, UK
[*]as2307@cam.ac.uk (corresponding author)
[+]am253@cam.ac.uk (corresponding author)

Supplementary material

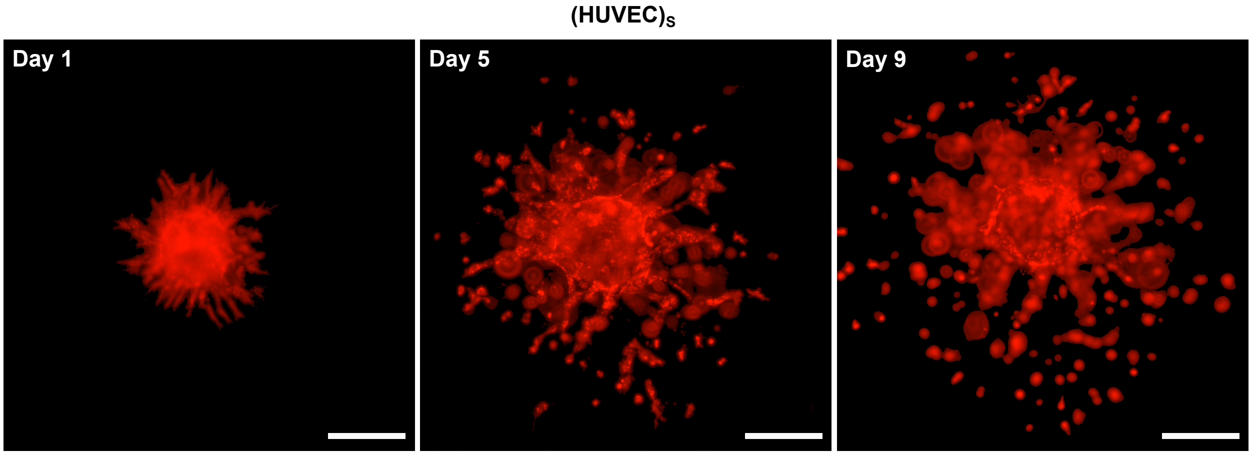

Figure 1. Fluorescence images showing the evolution over time (days 1, 5 and 9) of a HUVEC (red) spheroid embedded in a 7.5 mg/mL fibrin gel at 20% O_2. The scale bars represent 250 μm. Subscript S denotes cells seeded in the same spheroid.

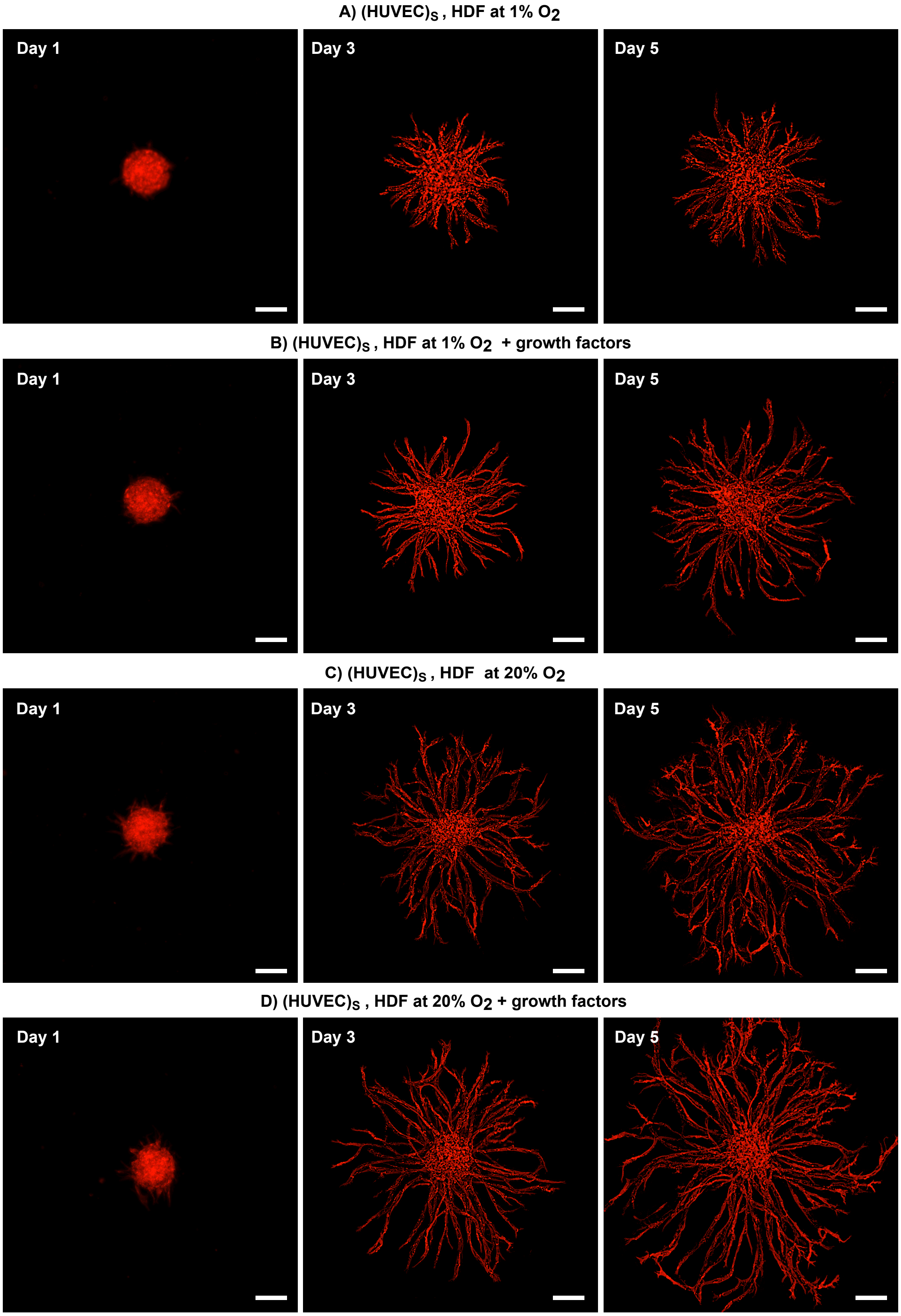

Figure 2. Fluorescence images showing the evolution over time (days 1, 3 and 5) of a HUVEC (red) spheroid surrounded by single HDF cells in a 7.5 mg/mL fibrin gel at (A) 1% O_2, (B) 1% O_2 with exogenous growth factors, (C) 20% O_2 and (D) 20% O_2 with exogenous growth factors. HDF cells are not stained so they are non-fluorescent. The scale bars represent 250 μm. Subscript S denotes cells seeded in the same spheroid.

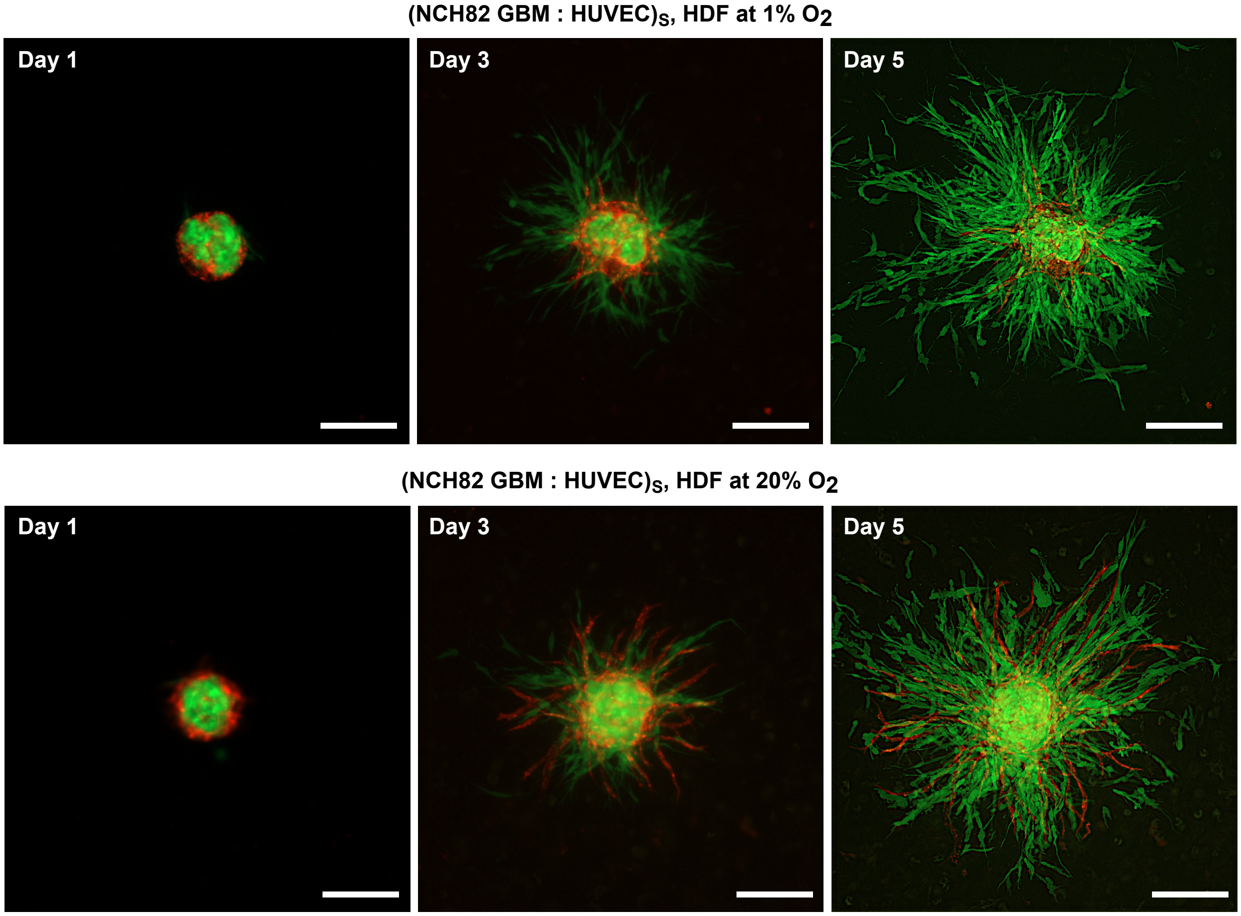

Figure 3. Fluorescence images showing the evolution over time (days 1, 3 and 5) of a NCH82 GBM (green) : HUVEC (red) spheroid surrounded by single HDF cells in a 7.5 mg/mL fibrin gel at 1% and 20% O_2. HDF cells are not stained so they are non-fluorescent. The scale bars represent 250 μm. Subscript S denotes cells seeded in the same spheroid.
